# H_2_S Protects Against Immobilization-Induced Muscle Atrophy via Reducing Oxidative Stress and Inflammation

**DOI:** 10.3389/fphys.2022.844539

**Published:** 2022-04-06

**Authors:** Miaomiao Xu, Xiaoguang Liu, Peng Bao, Yan Jie Wang, Jianqiang Lu, Yu Jian Liu

**Affiliations:** ^1^ School of Kinesiology, Shanghai University of Sport, Shanghai, China; ^2^ Guangzhou Sport University Sports and Health, Guangzhou Sport University, Guangzhou, China

**Keywords:** H2S, immobilization, muscle atrophy, fibrosis, inflammation, oxidative stress

## Abstract

Chronic inflammation and oxidative stress are major triggers of the imbalance between protein synthesis and degradation during the pathogenesis of immobilization-induced muscle atrophy. This study aimed to elucidate the effects of hydrogen sulfide (H_2_S), a gas transmitter with potent anti-inflammatory and antioxidant properties, on immobilization-induced muscle atrophy. Mice were allocated to control and immobilization (IM) groups, which were treated with slow (GYY4137) or rapid (NaHS) H_2_S releasing donors for 14 days. The results showed that both GYY4137 and NaHS treatment reduced the IM-induced muscle loss, and increased muscle mass. The IM-induced expressions of Muscle RING finger 1 (MuRF1) and atrogin-1, two muscle-specific E3 ubiquitin ligases, were decreased by administration of GYY4137 or NaHS. Both GYY4137 and NaHS treatments alleviated the IM-induced muscle fibrosis, as evidenced by decreases in collagen deposition and levels of tissue fibrosis biomarkers. Moreover, administration of GYY4137 or NaHS alleviated the IM-induced infiltration of CD45 ^+^ leukocytes, meanwhile inhibited the expressions of the pro-inflammatory biomarkers in skeletal muscles. It was found that administration of either GYY4137 or NaHS significantly attenuated immobilization-induced oxidative stress as indicated by decreased H_2_O_2_ levels and 8-hydroxy-2′-deoxyguanosine (8-OHdG) immunoreactivity, as well as increased total antioxidant capacity (T-AOC), nuclear factor erythroid-2-related factor 2 (NRF2) and NRF2 downstream anti-oxidant targets levels in skeletal muscles. Collectively, the present study demonstrated that treatment with either slow or rapid H_2_S releasing donors protected mice against immobilization-induced muscle fibrosis and atrophy. The beneficial effects of H_2_S on immobilization-induced skeletal muscle atrophy might be due to both the anti-inflammatory and anti-oxidant properties of H_2_S.

## Introduction

Long-term bed rest or unloading leads to muscle atrophy, which is associated with substantial prevalence and financial burden all over the world (Edwards et al., 2015). Muscle atrophy occurs when protein degradation rates exceed protein synthesis (Wall et al., 2013b). Two major protein degradation pathways, the proteasomal and the autophagic-lysosomal pathways, are activated during muscle atrophy. Growing evidence has shown that immobilization-induced muscle atrophy is a chronic inflammatory reaction (Komatsu et al., 2018), which is characterized by increases of pro-inflammatory biomarkers (Caron et al., 2011). Subsequently, the pro-inflammatory cytokines, such as TNF-α and IL-6, promote the muscle atrophy process by activating the ubiquitin-proteasome system, ultimately leading to muscle loss and functional impairment (Sente et al., 2016).

On the other hand, oxidative stress is recently considered as another major trigger of the imbalance between protein synthesis and degradation during the pathogenesis of immobilization-induced muscle atrophy (Pellegrino et al., 2011). Elevated production of ROS in disused skeletal muscles is found to activate both ubiquitin-proteasomal and the autophagic-lysosomal pathways, thus promoting muscle atrophy (Shenton et al., 2006). Notably, excessive ROS is also found to induce muscle atrophy through activating NF-κB pro-inflammatory signaling pathway (Zhao S et al., 2020). Taken together, these studies suggest that anti-inflammatory agents and/or antioxidants may be useful for the treatment of immobilization-induced muscle atrophy.

H_2_S, as a gas transmitter, is found to regulate inflammatory responses and oxidative/redox signaling pathways in a variety of tissues including different types of muscles (Chen et al., 2021). As shown in the literature, H_2_S donors exert complex and, at times, opposing effects such as pro-inflammatory or anti-inflammatory, pro-oxidant or anti-oxidant, cytotoxic or cytoprotective effects. One possible explanation for these discrepant data may be the different releasing patterns of H_2_S donors used in these various studies. To the best of our knowledge, the effects of H_2_S donors on immobilization-induced muscle atrophy remained unknown. Thus, the present study aimed to investigate and compare the effects of slow (GYY4137) and rapid (NaHS) H_2_S releasing donors on a mouse model of muscle atrophy induced by bilateral hindlimb immobilization. Our data showed that both slow and rapid H_2_S releasing donors protected against immobilization-induced muscle atrophy and fibrosis via reducing inflammation and oxidative stress.

## Methods

### Animals

Ten-week-old C57BL/6J male mice were obtained from the Model Animal Research Center of Nanjing University. Animals were housed in cages and given access to food and water ad libitum at a constant ambient temperature and humidity with 12 h day-night cycling. The experimental protocols were approved by the Ethics Review Committee for Animal Experimentation of the Shanghai University of Sport (102772020DW008).

### Immobilization-Induced Muscle Atrophy Model and Hydrogen Sulfide Donors Administration

We used an experimental model of immobilization-induced muscle atrophy. Briefly, mice were anesthetized via intraperitoneal injection of 35 mg/kg phenobarbital sodium. Both hindlimbs were shaved and wrapped in medical plaster with the knee extended, and the ankle positioned in plantar flexion to induce maximal atrophy of the both hindlimbs muscles (Frimel et al., 2005).

To evaluate the effect of slow H_2_S releasing donor GYY4137 on immobilization-induced muscle atrophy, mice were randomly divided into three groups: 1) Control group; 2) Immobilization group; 3) Immobilization + GYY4137 group. Mice in immobilization + GYY group were injected intraperitoneally with GYY4137 (Sigma, United States) at 50 mg/kg once daily for 2 weeks. The control and immobilization groups received identical doses of saline once daily for 2 weeks.

To evaluate the effect of rapid H_2_S releasing donor NaHS on immobilization-induced muscle atrophy, mice were randomly divided into three groups: 1) Control group; 2) Immobilization group; 3) Immobilization + NaHS group. Mice in immobilization + NaHS group were injected intraperitoneally with NaHS (Sigma, United States) at 1.12 mg/kg (20 μmol/kg) twice a day for 2 weeks. The control and immobilization groups received identical doses of saline twice a day for 2 weeks.

The chosen doses of GYY4137 (Huang et al., 2021) and NaHS (Smink et al., 2020) were based on previous studies and our preliminary experiments showing that GYY4137 and NaHS at the doses of 50 mg/kg and 20 μmol/kg were well tolerated in mice and significantly attenuated immobilization-induced muscle atrophy. Especially, in our preliminary experiments it was determined that the NAHS at the dose of 20 μmol/kg had a better protective effect than 50 μmol/kg in maintaining muscle mass and decreasing muscle atrophy related genes expressions (Supplementary Figure S1).

### Histological Stainings

Tibialis anterior (TA) muscle tissues were fixed with 4% paraformaldehyde overnight. The fixed tissue samples were then embedded in paraffin. Serial transverse sections of muscle tissues with 5 μm thickness were subjected to haematoxylin-eosin (HE) staining using commercial kit (KeyGEN, China) according to the instructions. Masson Trichromatic staining was performed with a Masson staining kit (Solarbio, China) according to the instructions.

### Quantitative Real-Time PCR

Briefly, total RNA samples were extracted from the TA muscle tissues using TRIzol (Invitrogen, United States). RNA samples were then reverse-transcribed into cDNA using a kit from Thermo Scientific (Applied Biosystems, United States). Relative expression levels of RNA transcripts were determined using gene-specific primers (Sangon Biotech, China), SYBR green (Vazyme Biotech, Nanjing, China). The expression of genes was normalized to β-actin and analyzed by the 2-ΔΔCT method. Gene-specific primers were listed in Table 1.

**TABLE 1:** Primers for real-time RT-PCR.

Target gene	Forward primer sequences	Reverse primer sequences
Col1a1	5′-TGG​TCC​TGC​TGG​TCC​TGC​TG-3′	5′-CTG​TCA​CCT​TGT​TCG​CCT​GTC​TC-3′
Col1a2	5′-AGG​TGG​CAA​AGG​TGA​ACA​AGG​C-3′	5′-GAC​CAG​CAG​GAC​CAG​GGA​GAC-3′
Col5a1	5′-CGA​AGT​TCC​TCA​GCC​GCA​GTG-3′	5′-GTC​ATA​GGC​AGC​TCG​GTT​GTC​AG-3′
Col14a1	5′-CAG​GCG​TCA​GGC​TTC​AGT​GAT​G-3′	5′-GTA​GGC​TGT​GGC​ATG​GCG​ATG-3′
PDGFRa	5′-CAG​GCA​GGG​CTT​CAA​CGG​AAC-3′	5′-GCG​TGC​GTC​CAT​CTC​CAG​ATT​C-3′
TGF-β	5′-TTCCTGGCGTTACCT TGGT-3′	5′-CCACTGCCG GACAACT-3′
α-SMA	5′-GGC​TTC​GCT​GGT​GAT​GAT​GCT​C-3′	5′-TCC​CTC​TCT​TGC​TCT​GGG​CTT​C-3′
IL-1β	5′-TGA​CGT​TCC​CAT​TAG​ACA​ACT​G-3′	5′-CCG​TCT​TTC​ATT​ACA​CAG​GAC​A-3′
IL-6	5′-GAA​CAA​CGA​TGA​TGC​ACT​TGC-3′	5′- CTT​CAT​GTA​CTC​CAG​GTA​GCT​ATG​GT-3′
TNF-α	5′-CCT​GTA​GCC​CAC​GTC​GTA​G-3′	5′-GGG​AGT​AGA​CAA​GGT​ACA​ACC​C-3′
NLRP3	5′-CGT​TGC​AAG​CTG​GCT​CAG​TA-3′	5′-GGG​GAC​TGG​GAT​ACA​GCC​TT-3′
CCL2	5′-GCT​CAG​CCA​GAT​GCA​GTT​AAC-3′	5′-CTC​TCT​CTT​GAG​CTT​GGT​GAC-3′
MuRF-1	5′-ATC​ACG​CAG​GAG​CAG​GAG​GAG-3′	5′-CTT​GGC​ACT​TGA​GAG​GAA​GGT​AGC-3′
Atrogin-1	5′-GTC​GGC​AAG​TCT​GTG​CTG​GTG-3′	5′-AGG​CAG​GTC​GGT​GAT​CGT​GAG-3′
NRF2	5′-TCT​CCT​CGC​TGG​AAA​AAG​AA-3′	5′-AAT​GTG​CTG​GCT​GTG​CTT​TA-3′
HO-1	5′-TCT​GGA​ATG​GAG​GGA​GAT​ACC-3′	5′-CAG​CAG​TCG​TGG​TCA​GTC​AA-3′
NQO1	5′-GCC​AAT​CAG​CGT​TCG​GTA​T-3′	5′-CCT​CCC​ATC​CTC​TCT​TCT​TCA-3′
MRP5	5′-TGG​GGG​ACT​ATG​CCG​CTA​T-3′	5′-AGA​AAT​TCT​GCA​CCC​CAG​CC-3′
ARK1B10	5′-ACC​CGC​TCC​TAC​TGA​CTC​CT-3′	5′-ACA​TTG​CCC​TTT​GCT​GAG​AT-3′
β-actin	5′-TGG​AAG​GTG​GAC​AGT​GAG​GC-3′	5′-CCC​AGG​CAT​TGC​TGA​CAG​G-3′

### Measurements of Total Antioxidant Capacity and H_2_O_2_

The total antioxidant capacity (T-AOC) and H_2_O_2_ content in the gastrocnemius (GAS) muscle tissue homogenates were detected according to the manufacturer’s instructions (Westang, Shanghai, China). All results were standardized by the protein concentration of muscle homogenates.

### Immunofluorescence

Paraffin sections (4 μm) of TA muscle tissues were rehydrated and microwaved in citric acid buffer to retrieve antigens. After incubation with 10% BSA for 1 h, the sections were respectively incubated with primary antibodies against CD45 (1:200; Abcam, MA, United States) and 8-hydroxy-2′-deoxyguanosine (8-OHdG) (1:200; Abcam) overnight. Subsequently, tissue slices were incubated with secondary antibody corresponding to each primary antibodies in dark for 1 h and counter-stained with 4′,6-diamidino-2-phenylindole (DAPI) (Beyotime, Jiangsu, China). Stained slices were observed using a laser scanning confocal microscopy.

### Western Blotting

TA muscles were homogenized in cold RIPA buffer (Beyotime, Jiangsu, China) by an ultrasonic vibrator and a mechanical homogenizer. Proteins (30 mg) were separated by 10% SDS–PAGE and subsequently transferred to polyvinylidene difluoride membranes (Millipore Corp., Bedford, MA, United States). After being blocked, immunoblots were incubated with primary antibody against anti-nuclear factor erythroid-2-related factor 2 (NRF2) (1:1,000; Santa Cruz Biotechnologies), anti-p-AKT (1:1,000; ProteinTech Group, Wuhan, China), anti-AKT (1:1,000; ProteinTech Group), and anti-β-actin (1:10,000; Bioworld, Nanjing, China) at 4 s°C overnight, followed by incubation with a secondary horseradish peroxidase-conjugated IgG (1:5,000; Abcam) for 1 h at room temperature. Immunoreactive proteins were visualized using the enhanced chemiluminescence Western blotting detection system (Millipore). Staining intensity of the bands was measured using a densitometer (Syngene, Braintree, United Kingdom) together with Genesnap and Genetools software (Syngene).

### Statistical Analysis

Data were presented as mean ± S.E.M. The comparison among multiple groups was performed by one-way ANOVA followed by a Bonferroni correction post-hoc test. Statistical significance was set at *p* < 0.05. All statistical analyses were done with SPSS 22.0 (SPSS Inc., Chicago, United States).

## Results

### The Effects of Slow (GYY4137) and Rapid (NaHS) H_2_S Releasing Donors on Immobilization-Induced Muscle Atrophy

**FIGURE 1:**
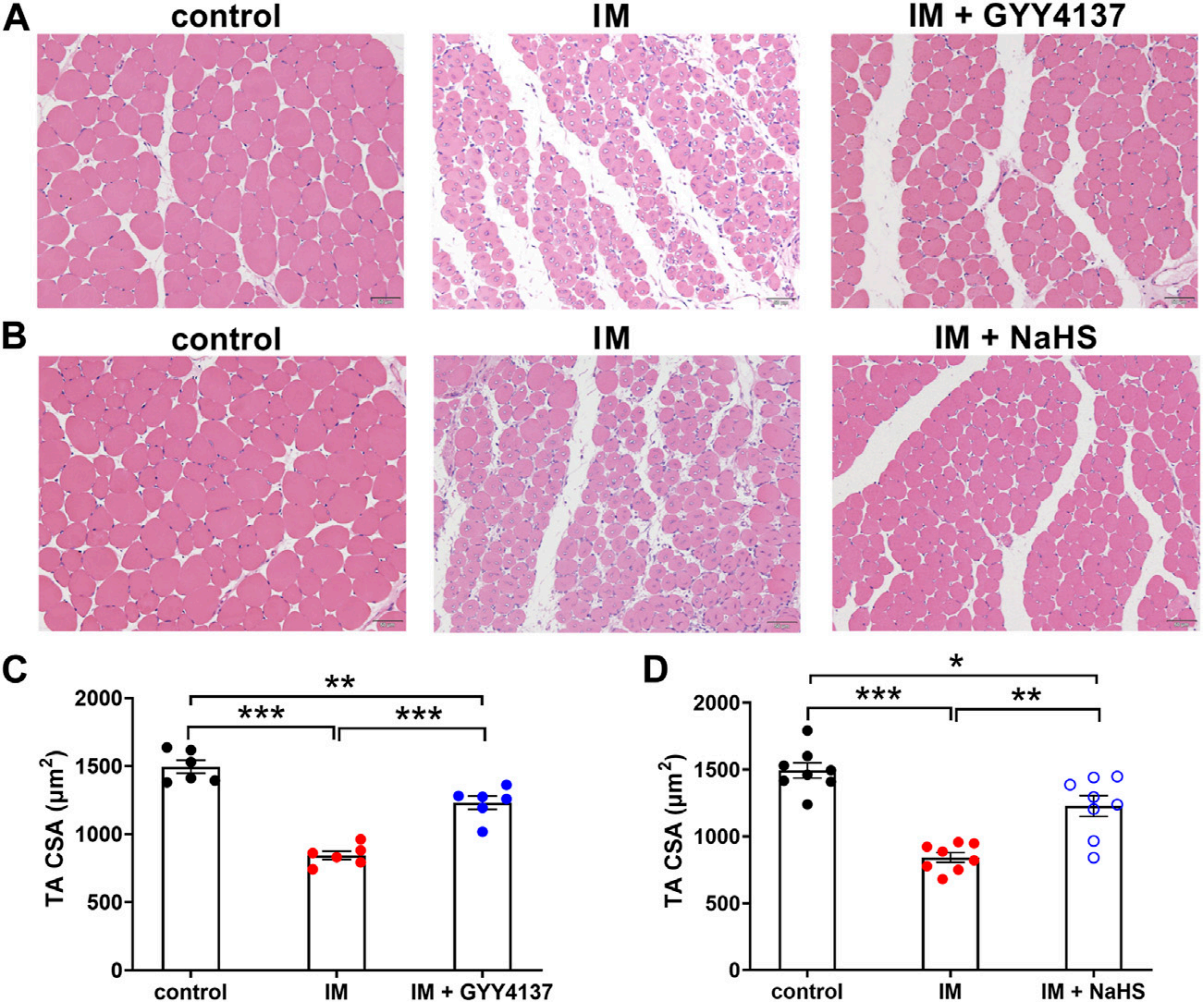
H2S donors alleviate the immobilization (IM)-induced muscle atrophy. Mice were subjected to IM and treated with GYY4137 **(A)**, 50 μg/kg/d, ip), or NaHS **(B)**, 1.12 mg/kg, twice a day, ip) for 2 weeks. The control and IM groups received identical doses of saline for 2 weeks. A & b, Representative photomicrographs of H&E-stained TA muscles (scale bars, 50 μm). **(C)**, Quantification of fiber CSAs of TA muscle in control, IM, and IM + GYY4137 mice (n = 6 in each group). **(D)**, Quantification of fiber CSAs of TA muscle in control, IM, and IM + NaHS mice (n = 8 in each group). Data represent means ± SEM. *p < 0.05, **p < 0.01, ***p < 0.001.

**FIGURE 2:**
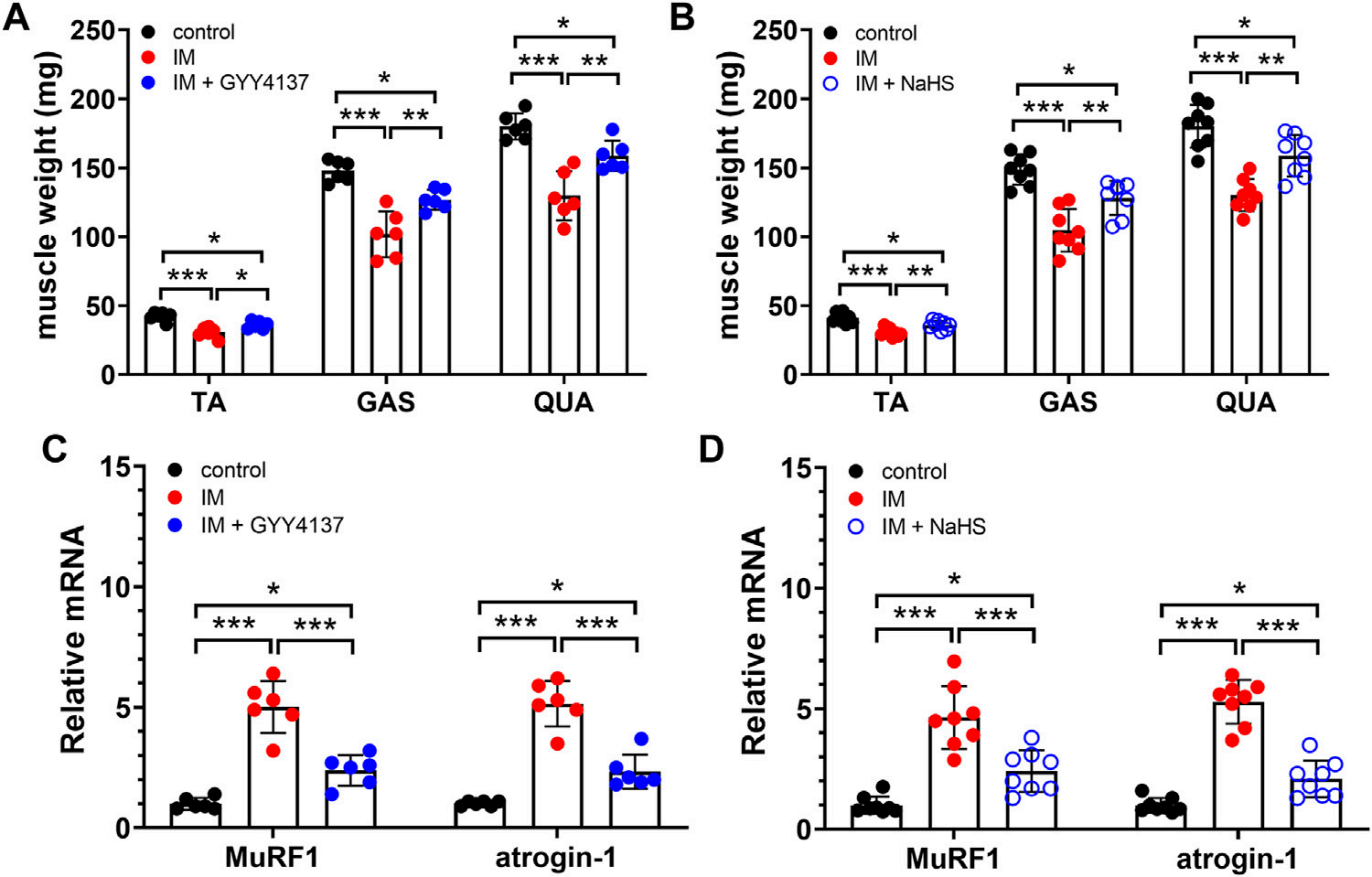
H2S donors protect the IM-induced muscle loss and downregulate muscle atrophy-related genes expression. Mice were subjected to IM and treated with GYY4137 (a and c, 50 μg/kg/d, ip), or NaHS (b and d, 1.12 mg/kg, twice a day, ip) for 2 weeks. The control and IM groups received identical doses of saline for 2 weeks. **(A)**, TA, GAS, and QUA muscle weights in control, IM, and IM + GYY4137 mice (n = 6 in each group). **(B)**, TA, GAS, and QUA muscle weights in control, IM, and IM + NaHS mice (n = 8 in each group). **(C)**, Expression of MuRF1 and atrogin-1 in TA muscles in control, IM, and IM + GYY4137 mice (n = 6 in each group). **(D)**, Expression of MuRF1 and atrogin-1 in TA muscles in control, IM, and IM + NaHS mice (n = 8 in each group). Data represent means ± SEM. *p < 0.05, **p < 0.01, ***p < 0.001.

Muscle atrophy is characterized by muscle fibers size decrease and muscle mass loss. We first examined the effect of H_2_S donor GYY4137 and NaHS on cross-sectional areas (CSAs) of skeletal muscle fibers by using H&E staining. Results of the staining showed a significant reduction of CSAs in TA muscle isolated from immobilization group mice in comparison to control group mice (Figure 1). Additionally, we did notice an improvement after 2 weeks of either GYY4137 or NaHS treatment (Figures 1A–D). We also measured the wet weights of the tibialis anterior (TA), gastrocnemius (GAS), and quadriceps (QUA) muscles. In immobilization group, the weights of skeletal muscles were significantly lower than those in control group. Both GYY4137 and NaHS treatment significantly reversed the immobilization-induced loss of skeletal muscle mass (Figures 2A,B).

MuRF1 and atrogin-1, two muscle-specific E3 ubiquitin ligases, are well-known to be upregulated in skeletal muscle under atrophy-inducing conditions, thus serve as sensitive biomarkers related to muscle wasting. In consistent with previous studies, we found that both MuRF1 and atrogin-1 were significantly upregulated in skeletal muscles obtained from immobilization mice as compared to those obtained from control mice. Moreover, the immobilization-induced mRNA expressions of MuRF1 and atrogin-1 in skeletal muscles were significantly decreased by administration of GYY4137 and NaHS (Figures 2C,D). Taken together, these results suggesting that both slow and rapid H2S releasing donors attenuated H2S treatment alleviated immobilization-induced muscle atrophy.

### The Effects of Slow (GYY4137) and Rapid (NaHS) H_2_S Releasing Donors on Immobilization-Induced Muscle Fibrosis

**FIGURE 3:**
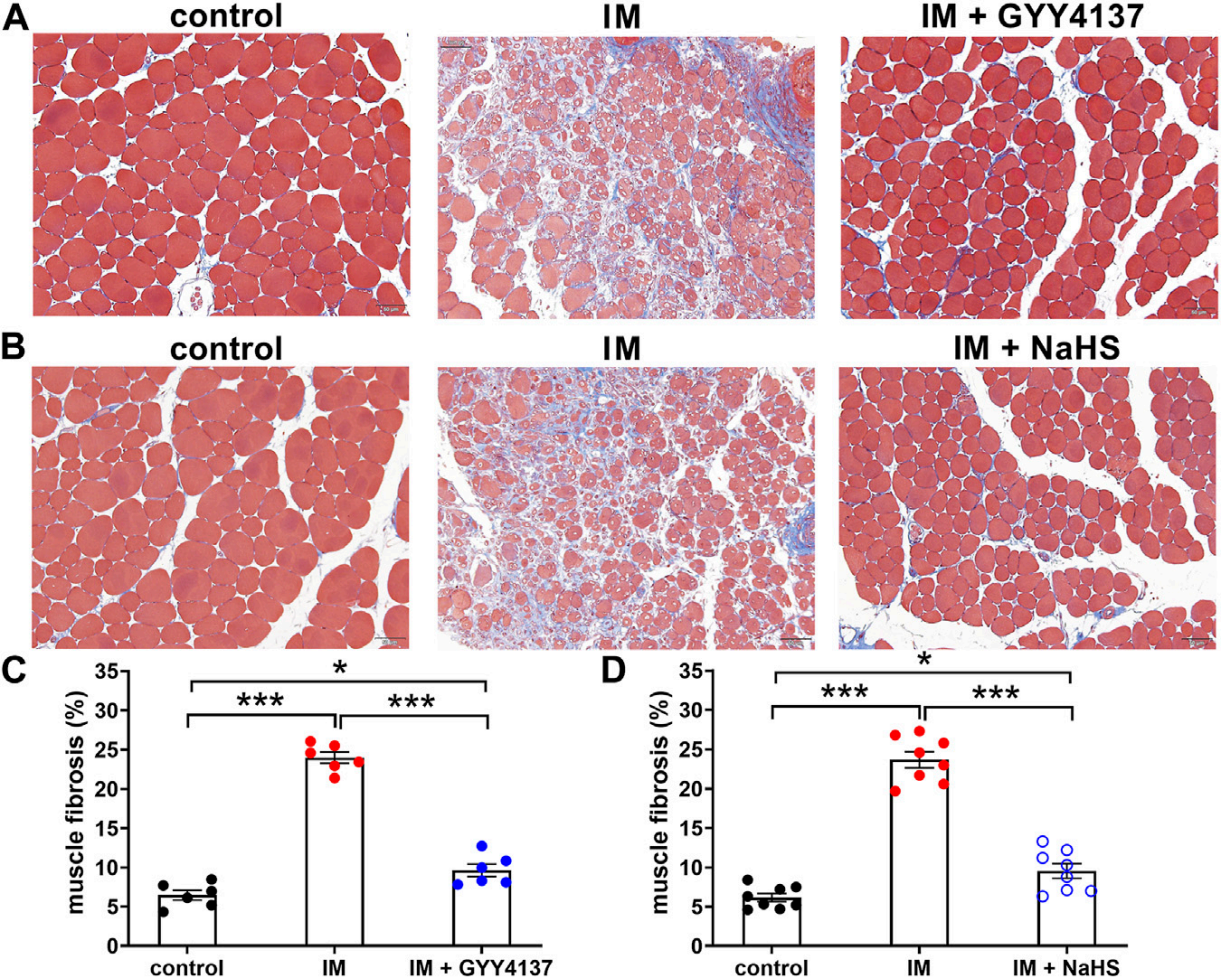
H2S donors reduce the IM-induced muscle fibrosis. Mice were subjected to IM and treated with GYY4137 (a and c, 50 μg/kg/d, ip), or NaHS (b and d, 1.12 mg/kg, twice a day, ip) for 2 weeks. The control and IM groups received identical doses of saline for 2 weeks. **(A)** and **(B)**, Representative photomicrographs of Masson-stained TA muscles (scale bars, 50 μm). **(C)**, Quantification of the fibrosis fraction of TA muscles in control, IM, and IM + GYY4137 mice (n = 6 in each group). **(D)**, Quantification of the fibrosis fraction of TA muscles in control, IM, and IM + NaHS mice (n = 8 in each group). Data represent means ± SEM. *p < 0.05, **p < 0.01, ***p < 0.001.

**FIGURE 4:**
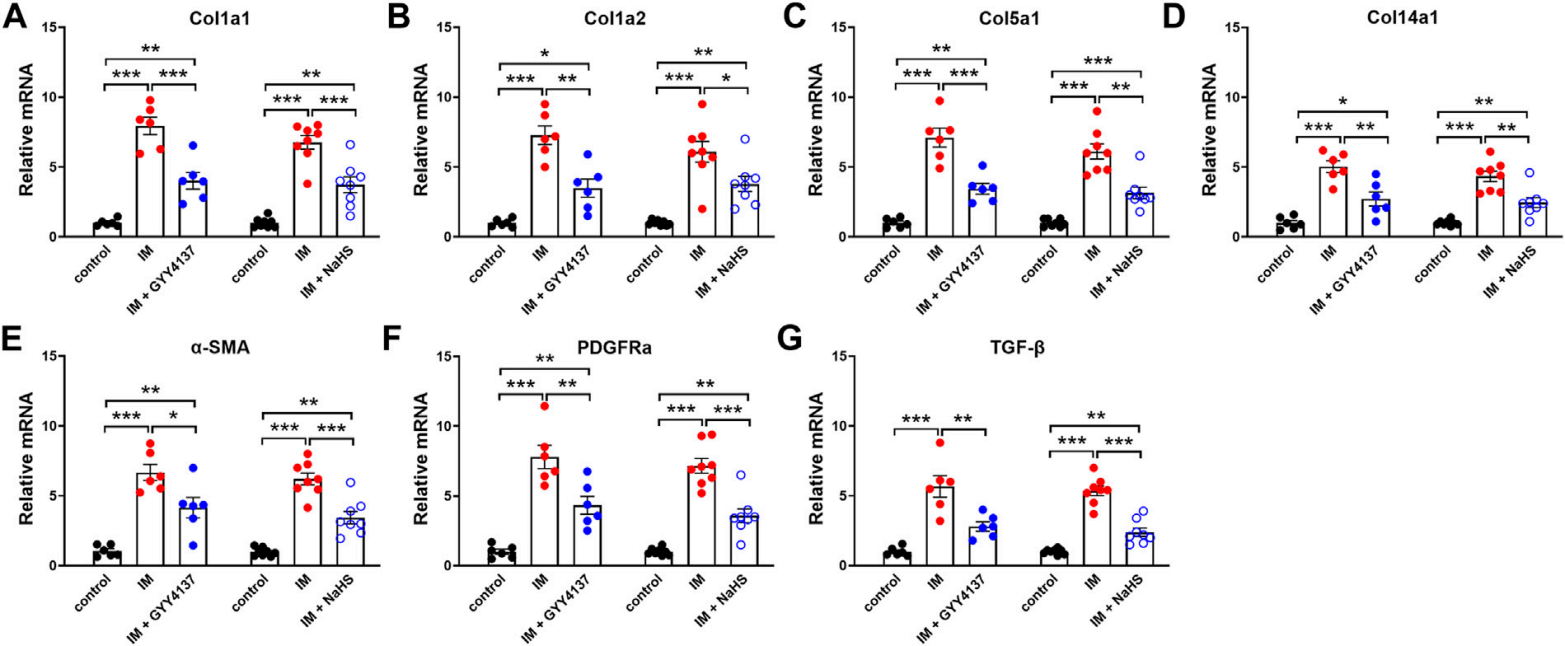
H2S donors downregulate the IM-induced mNRA expressions of tissue fibrosis biomarkers in skeletal muscles. Mice were subjected to IM and treated with GYY4137 (50 μg/kg/d, ip, n = 6), or NaHS (1.12 mg/kg, twice a day, ip, n = 8) for 2 weeks. The control and IM groups received identical doses of saline for 2 weeks. **(A-G)** showed mRNA expressions of tissue fibrosis biomarkers including Col1a1 **(A)**, Col1a2 **(B)**, Col5a1 **(C)**, Col14a1 **(D)**, α-SMA **(E)**, PDGFRa **(F)**, and TGF-β **(G)** in TA muscles. Data represent means ± SEM. *p < 0.05, **p < 0.01, ***p < 0.001.

Muscle fibrosis is the main cause of reduced muscle extensibility and decreased range of motion in immobilization-induced skeletal muscle atrophy. As shown in Figures 2, 3 weeks after immobilization, Masson’s trichrome staining showed a significant increase in collagen deposition in the skeletal muscle. The collagen deposition appeared diffusively in the interstitium of skeletal muscle, and was accompanied by cellular infiltrates. The extensive deposition of fibrillar collagen and the destruction of normal skeletal muscle architecture observed in immobilization-treated mice were markedly attenuated by administration of either GYY4137 or NaHS (Figures 3A–D). In addition, we also examined the mRNA expressions of tissue fibrosis biomarkers including Col1a1, Col1a2, Col5a1, Col14a1, α-SMA, PDGFRa, and TGF-β. As expected, the mRNA levels of these tissue fibrosis biomarkers were significantly increased in the immobilization group as compared to the control group. Administration of either GYY4137 or NaHS significantly inhibited the immobilization-induced mRNA expressions of Col1a1, Col1a2, Col5a1, Col14a1, α-SMA, PDGFRa, and TGF-β (Figures 4A–G). Taken together, these results indicated that both slow and rapid H_2_S releasing donors attenuated immobilization-induced skeletal muscle fibrosis.

### The Effects of Slow (GYY4137) and Rapid (NaHS) H_2_S Releasing Donors on Immobilization-Induced Inflammatory Responses in Skeletal Muscle.

**FIGURE 5:**
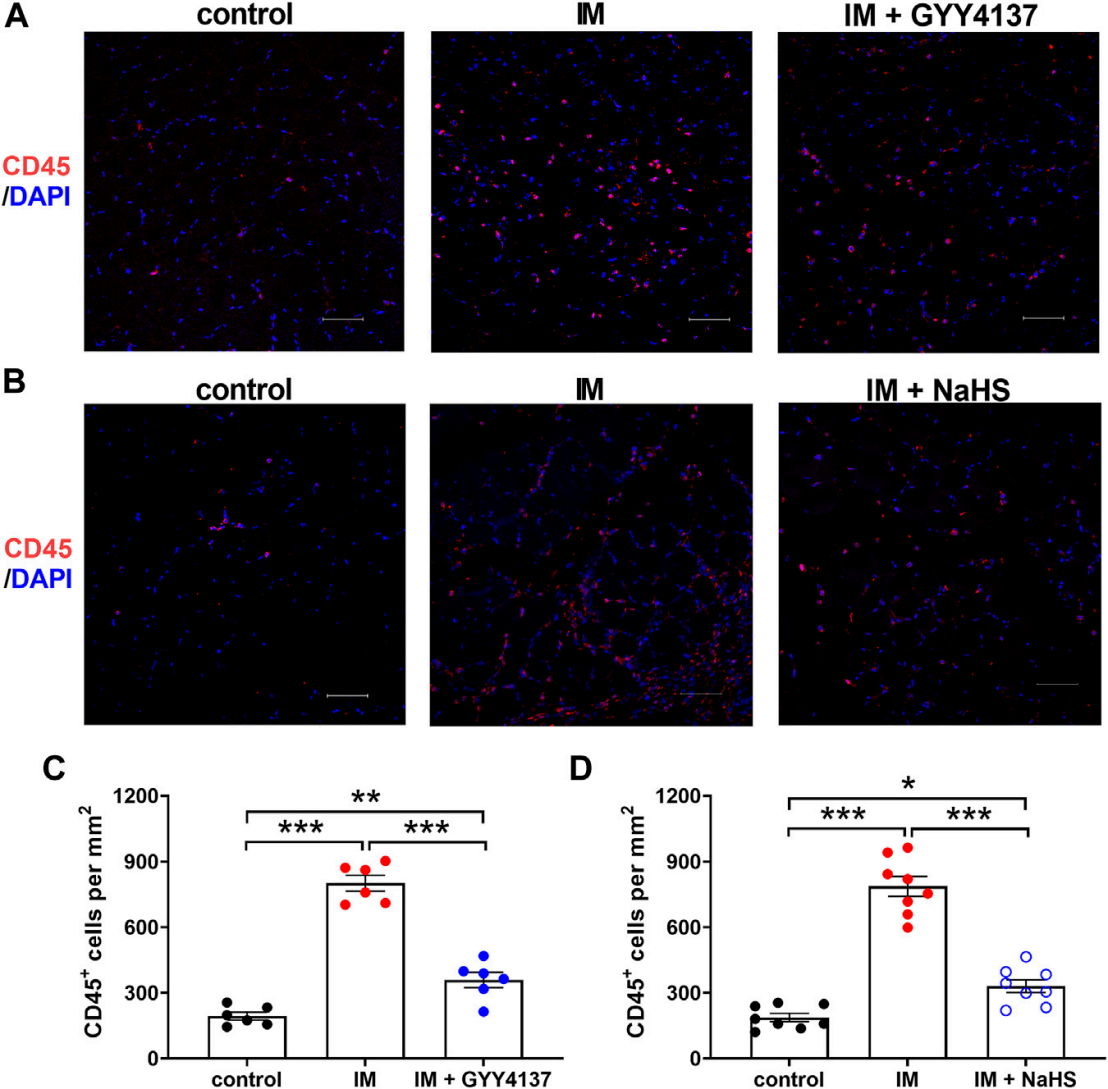
H2S donors alleviate the IM-induced leukocyte infiltration in skeletal muscles. Mice were subjected to IM and treated with GYY4137 (a and c, 50 μg/kg/d, ip), or NaHS (b and d, 1.12 mg/kg, twice a day, ip) for 2 weeks. The control and IM groups received identical doses of saline for 2 weeks. **(A)** and **(B)**, Representative micrographs of CD45 ^+^ leukocyte staining in TA muscles (Scale bar, 50 μm). **(C)**, Quantitative analysis of CD45 positive staining leukocytes of TA muscles in control, IM, and IM + GYY4137 mice (n = 6 in each group). **(D)**, Quantitative analysis of CD45 positive staining leukocytes of TA muscles in control, IM, and IM + NaHS mice (n = 8 in each group). Data represent means ± SEM. *p < 0.05, **p < 0.01, ***p < 0.001.

**FIGURE 6:**
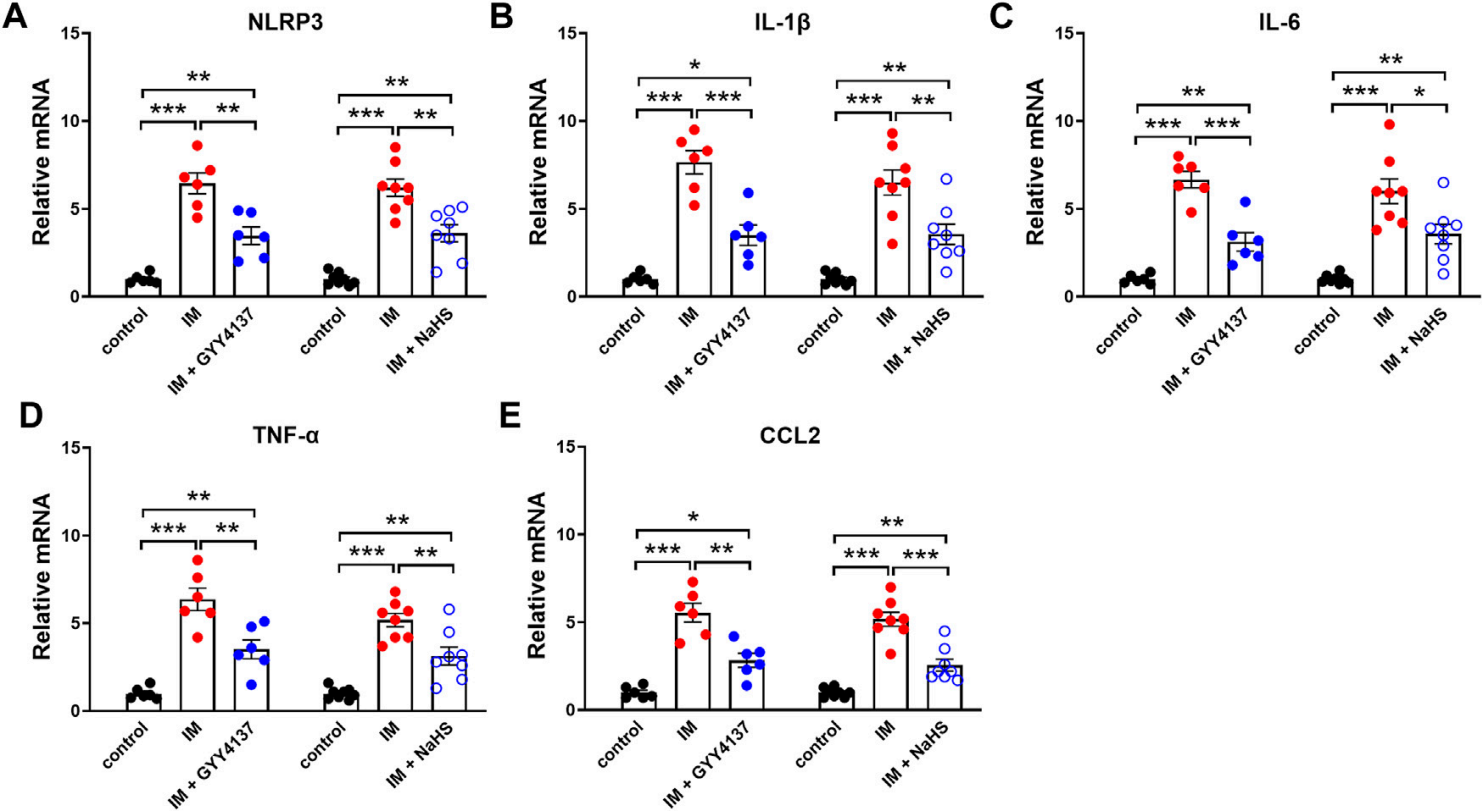
H2S donors inhibit the IM-induced mNRA expressions of pro-inflammatory biomarkers in skeletal muscles. Mice were subjected to IM and treated with GYY4137 (50 μg/kg/d, ip, n = 6), or NaHS (1.12 mg/kg, twice a day, ip, n = 8) for 2 weeks. The control and IM groups received identical doses of saline for 2 weeks. a-e showed mRNA expressions of pro-inflammatory biomarkers including NLRP3 **(A)**, IL-1β **(B)**, IL-6 **(C)**, TNF-α **(D)**, and CCL2 **(E)** in TA muscles. Data represent means ± SEM. *p < 0.05, **p < 0.01, ***p < 0.001.

Chronic inflammatory reaction has been recognized as an important trigger of immobilization-induced muscle atrophy and fibrosis. We then investigated the effects of H2S donor GYY4137 and NaHS on leukocyte infiltration and the mRNA expressions of the pro-inflammatory biomarkers including NLRP3, IL-1β, IL-6, TNF-α, and CCL-2 in skeletal muscles. As shown in Figure 5, there were very few CD45-positive staining cells in the skeletal muscle tissues of the Control group. Mice that underwent immobilization operation developed severe infiltration of CD45 ^+^ leukocytes. The quantification analysis showed that the percentage of CD45 ^+^ leukocytes in skeletal muscles was significantly decreased by administration of either GYY4137 or NaHS (Figures 5A–D). Immobilization also caused significant increases in mRNA levels of NLRP3, IL-1β, IL-6, TNF-α, and CCL-2, which were significantly inhibited by GYY4137 or NaHS (Figures 6A–E). These data indicated that both slow and rapid H_2_S releasing donors attenuated immobilization-induced inflammatory responses in skeletal muscle.

### The Effects of Slow (GYY4137) and Rapid (NaHS) H_2_S Releasing Donors on Immobilization-Induced Oxidative Stress in Skeletal Muscle

**FIGURE 7:**
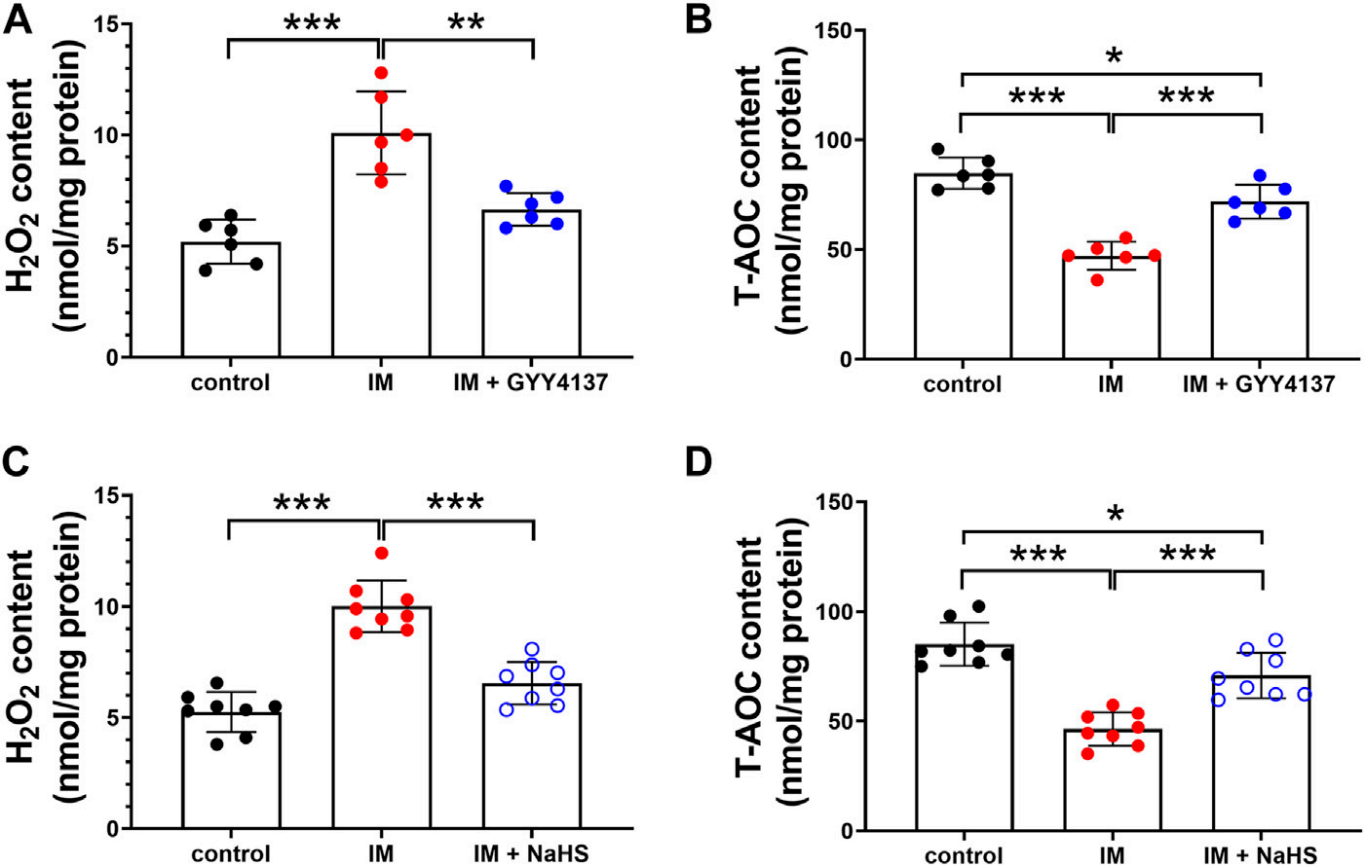
H2S donors repress the IM-induced oxidative stress in skeletal muscles. Mice were subjected to IM and treated with GYY4137 (a and b, 50 μg/kg/d, ip), or NaHS (c and d, 1.12 mg/kg, twice a day, ip) for 2 weeks. The control and IM groups received identical doses of saline for 2 weeks. **(A)**, The level of H_2_O_2_ in GAS muscle homogenates in control, IM, and IM + GYY4137 mice (n = 6 in each group). **(B)**, The level of T-AOC in GAS muscle homogenates in control, IM, and IM + GYY4137 mice (n = 6 in each group). **(C)**, The level of H_2_O_2_ in GAS muscle homogenates in control, IM, and IM + NaHS mice (n = 8 in each group). **(D)**, The level of T-AOC in GAS muscle homogenates in control, IM, and IM + NaHS mice (n = 8 in each group). Data represent means ± SEM. *p < 0.05, **p < 0.01, ***p < 0.001.

**FIGURE 8:**
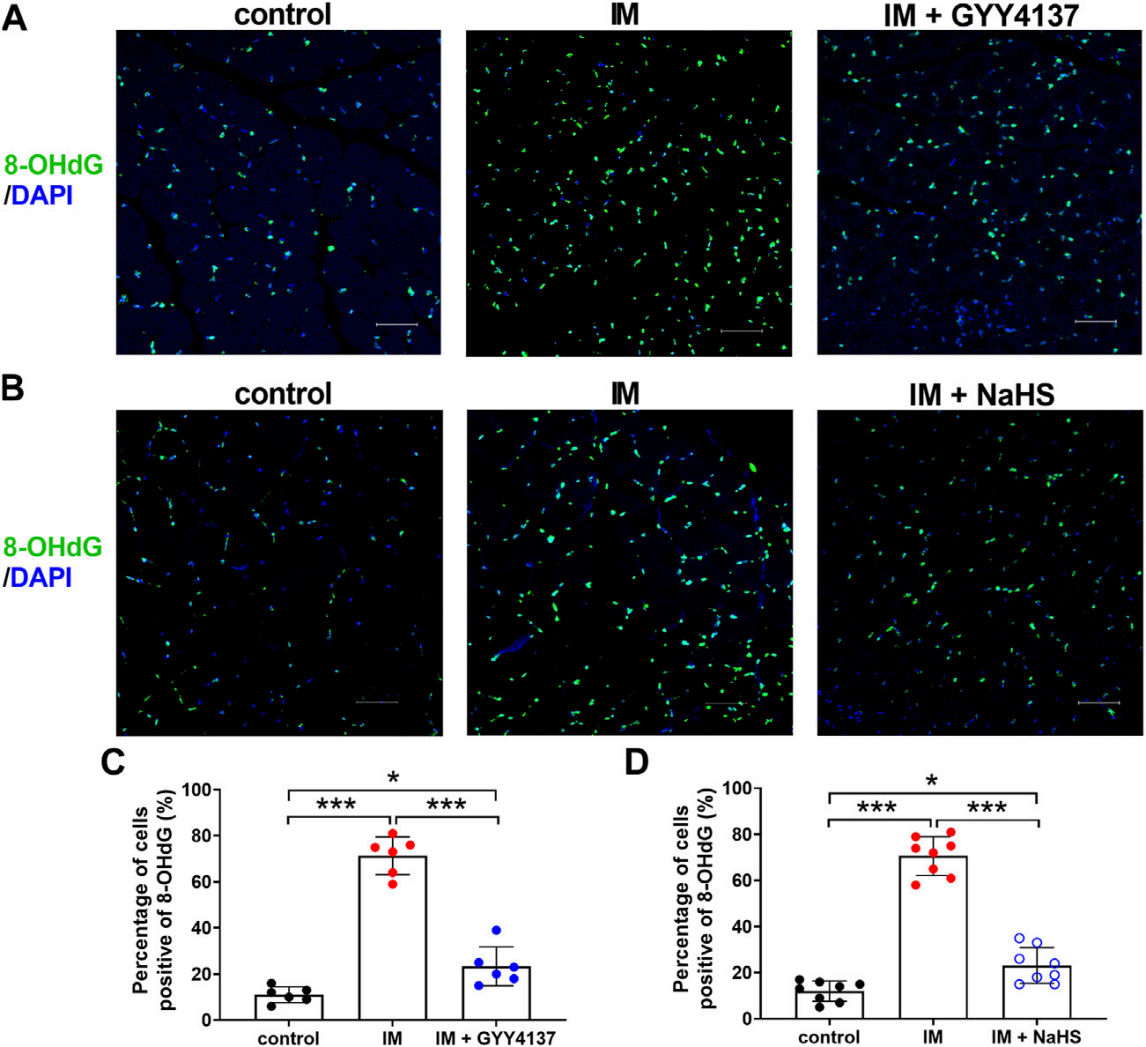
H2S donors attenuate the IM-induced muscle 8-OHdG positive staining. Mice were subjected to IM and treated with GYY4137 (a and c, 50 μg/kg/d, ip), or NaHS (b and d, 1.12 mg/kg, twice a day, ip) for 2 weeks. The control and IM groups received identical doses of saline for 2 weeks. **(A)** and **(B)**, Representative micrographs of 8-OHdG staining in TA muscles (Scale bar, 50 μm). **(C)**, Quantitative analysis of 8-OHdG positive staining of TA muscles in control, IM, and IM + GYY4137 mice (n = 6 in each group). **(D)**, Quantitative analysis of 8-OHdG positive staining TA muscles in control, IM, and IM + NaHS mice (n = 8 in each group). Data represent means ± SEM. *p < 0.05, **p < 0.01, ***p < 0.001.

Oxidative stress is recently considered as another major trigger of the imbalance between protein synthesis and degradation in skeletal muscle. We then investigated the effects of H_2_S donor GYY4137 and NaHS on immobilization-induced oxidative stress. As shown in Figures 7A–D, immobilization significantly increased H_2_O_2_ levels, whereas decreased T-AOC levels in skeletal muscles. In addition, 8-OHdG immunoreactivity, an index of oxidative DNA damage, was also profoundly increased in immobilization group as compared with control group (Figures 8A–D). It was found that administration of either GYY4137 or NaHS significantly attenuated immobilization-induced oxidative stress as indicated by decreased H_2_O_2_ levels and 8-OHdG immunoreactivity, as well as increased T-AOC levels in skeletal muscles.

### The Effects of Slow (GYY4137) and Rapid (NaHS) H_2_S Releasing Donors on Immobilization-Induced Changes in NRF2 and NRF2 Downstream Targets in Skeletal Muscle

**FIGURE 9:**
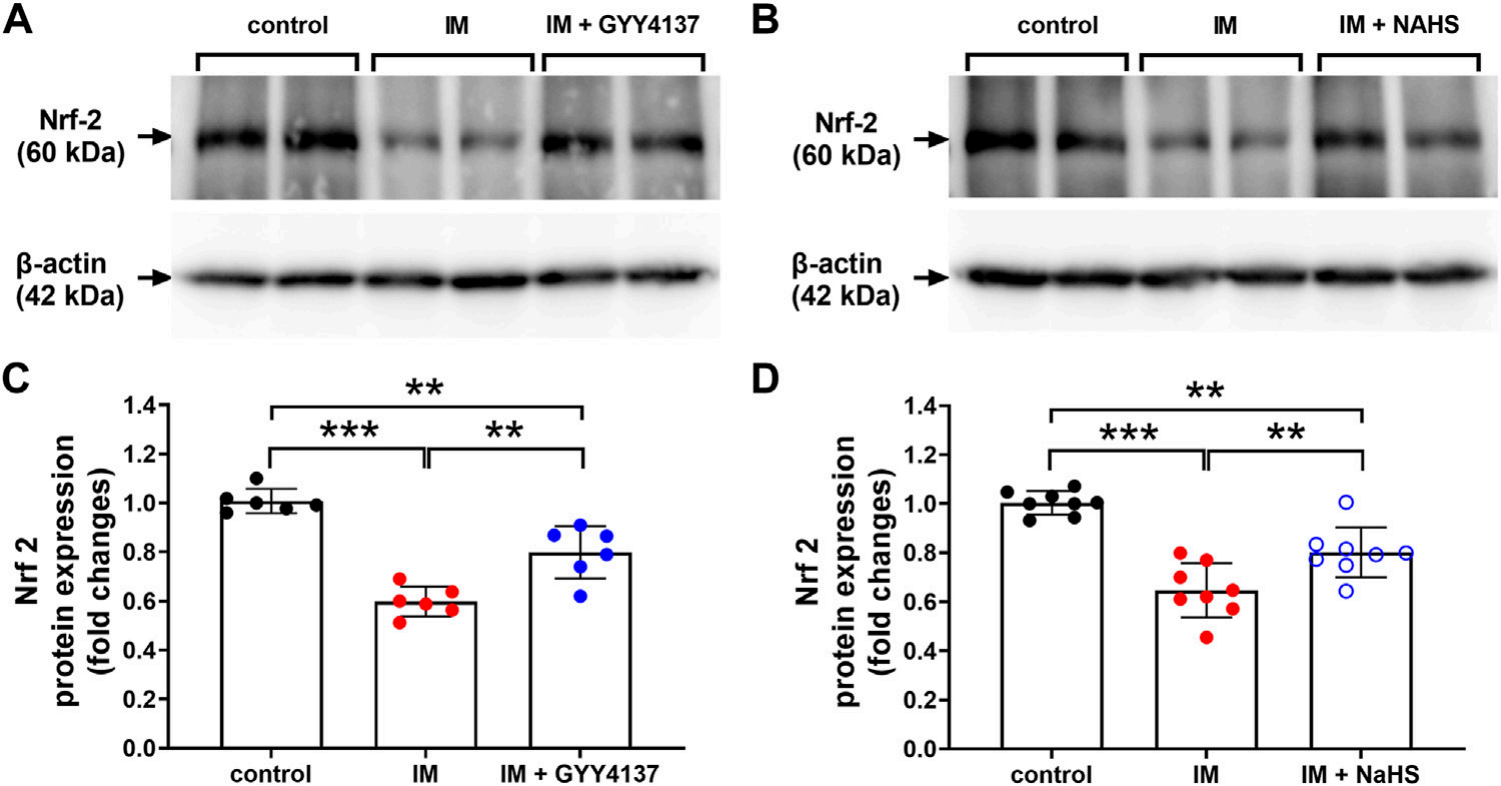
H2S donors improve the IM-induced changes in NRF2 protein expression in skeletal muscle. Mice were subjected to IM and treated with GYY4137 (a and c, 50 μg/kg/d, ip), or NaHS (b and d, 1.12 mg/kg, twice a day, ip) for 2 weeks. The control and IM groups received identical doses of saline for 2 weeks. **(A)** & **(C)**, NRF2 protein expression by Western blot analysis of TA muscle in control, IM, and IM + GYY4137 mice (n = 6 in each group). **(B)** & **(D)**, NRF2 protein expression by Western blot analysis of TA muscle in control, IM, and IM + NaHS mice (n = 8 in each group). Data represent means ± SEM. *p < 0.05, **p < 0.01, ***p < 0.001.

**FIGURE 10:**
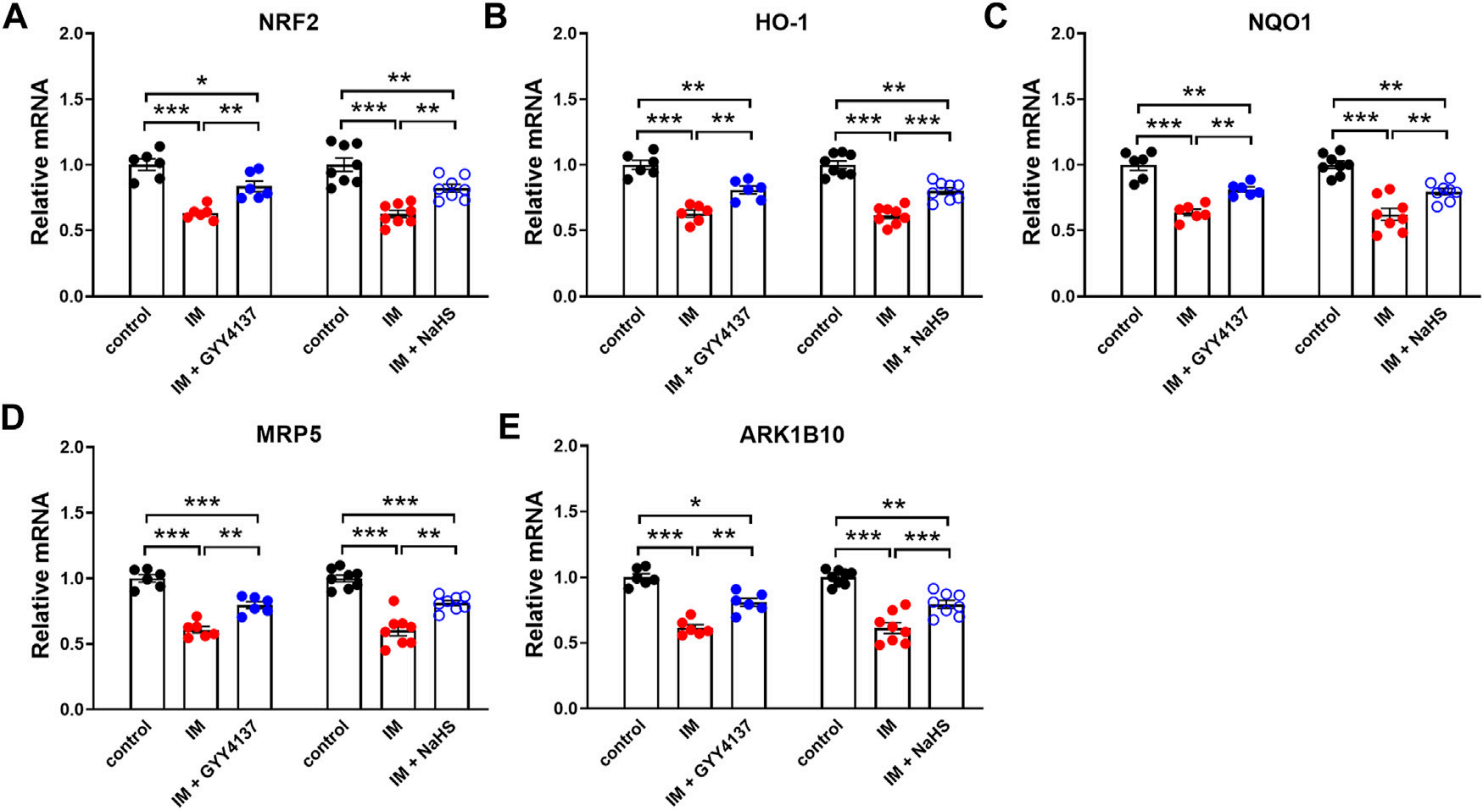
H2S donors protect the IM-induced changes in the mRNA levels of NRF2 and NRF2 downstream target genes in skeletal muscle. Mice were subjected to IM and treated with GYY4137 (50 μg/kg/d, ip, n = 6), or NaHS (1.12 mg/kg, twice a day, ip, n = 8) for 2 weeks. The control and IM groups received identical doses of saline for 2 weeks. a-e showed mRNA expressions of NRF2 **(A)** and NRF2 downstream target genes including HO-1 **(B)**, NQO1 **(C)**, MRP5 **(D)**, and ARK1B10 **(E)** in TA muscles. Data represent means ± SEM. *p < 0.05, **p < 0.01, ***p < 0.001.

NRF2 has recently emerged as a potential target of associated oxidative-stress regulation (Baumfalk et al., 2021). Previous study has reported NRF2 contributed to the protective effect of H_2_S against oxidative stress in a denervation induced muscle atrophy model (Kuo et al., 2015). We next examined whether NRF2 was involved in the protective effects of slow (GYY4137) and rapid (NaHS) H_2_S releasing donors. In this study, we found that IM inhibited NRF2 protein and mRNA expression in TA muscle (Figure 9 and Figure10A). Both slow (GYY4137) and rapid (NaHS) H_2_S releasing donors profoundly reversed IM-induced downregulation of NRF2 protein and mRNA expression (Figures 9A–D and Figure 10A). We also measured the mRNA levels of four NRF2 downstream target anti-oxidant and cytoprotective genes, heme oxygenase-1 (HO-1), NAD(P)H dehydrogenase quinone 1 (NQO1), multidrug-resistance-associated protein 5 (MRP5), and aldo–keto reductase 1B10 (AKR1B10). As shown in Figures 10B–E, IM led to significant decreases in HO-1, NQO1, MRP5, and AKR1B10 expression in TA muscle. Both slow (GYY4137) and rapid (NaHS) H_2_S releasing donors treatments led to significant increases in NRF2 downstream target anti-oxidant and cytoprotective genes expression compared with IM group (Figures 10B–E).

### Either Slow (GYY4137) or Rapid (NaHS) H_2_S Releasing Donors had No Significant Effect on the Expression of p-AKT/AKT Protein in Skeletal Muscle

**FIGURE 11:**
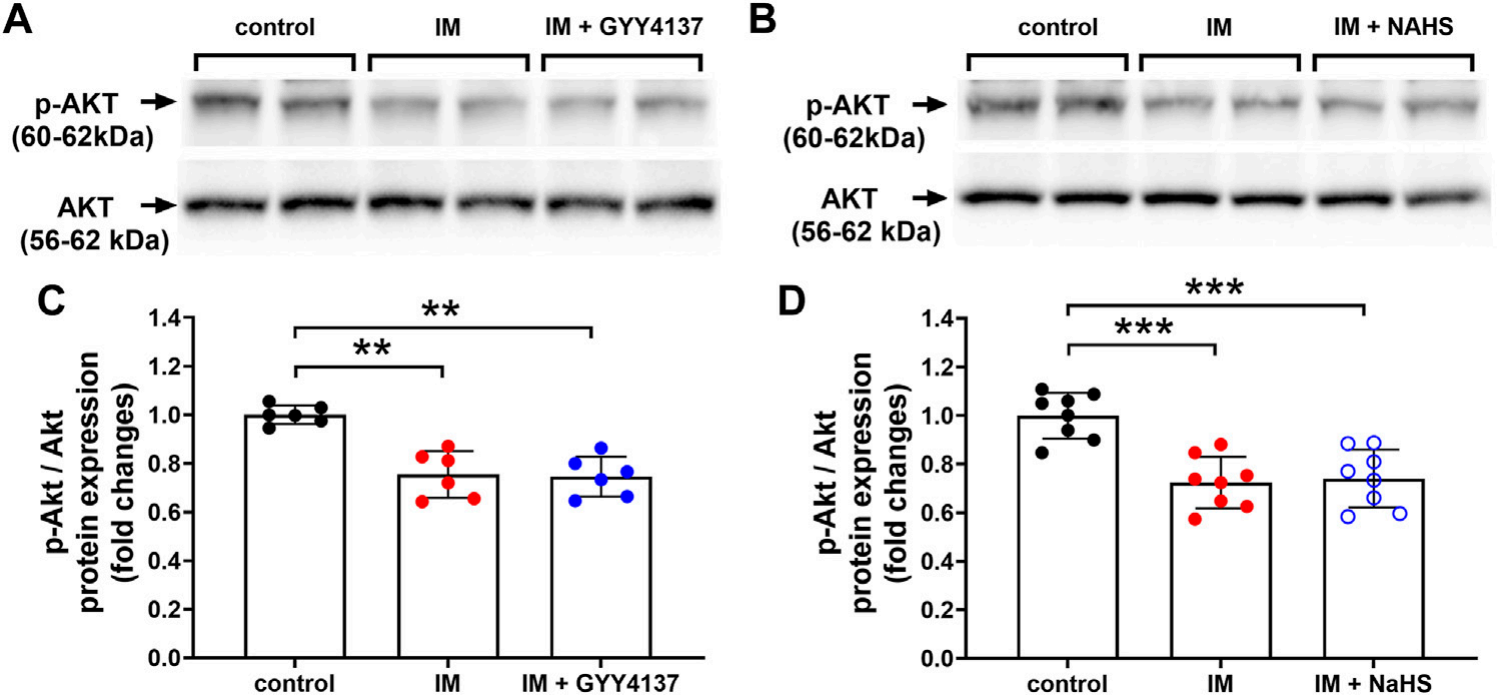
H2S donors have no effect on p-AKT/AKT protein expression in skeletal muscle. Mice were subjected to IM and treated with GYY4137 (a and c, 50 μg/kg/d, ip), or NaHS (b and d, 1.12 mg/kg, twice a day, ip) for 2 weeks. The control and IM groups received identical doses of saline for 2 weeks. **(A)** & **(C)**, p-AKT/AKT protein expression by Western blot analysis of TA muscle in control, IM, and IM + GYY4137 mice (n = 6 in each group). **(B)** & **(D)**, p-AKT/AKT protein expression by Western blot analysis of TA muscle in control, IM, and IM + NaHS mice (n = 8 in each group). Data represent means ± SEM. *p < 0.05, **p < 0.01, ***p < 0.001.

Numerous studies have shown that H_2_S can activate AKT and participate in a variety of tissue-protecting effects of H_2_S (Bitar et al., 2018). We next examined whether AKT was involved in the protective effects of slow (GYY4137) and rapid (NaHS) H_2_S releasing donors. In this study, we found that IM decreased p-AKT/AKT protein expression in TA muscle. However, either GYY4137 (slow releasing) or NAHS (rapid releasing) H_2_S donors had no significant effect on the expression of p-AKT/AKT protein (Figure 11).

## Discussion

Both chronic inflammatory reaction and oxidative stress have been already proposed in the pathogenesis of skeletal muscle atrophy due to different etiologies (Moylan and Reid, 2007; Meng and Yu, 2010). Previous studies have shown that administration of H_2_S donor NaHS can alleviate skeletal muscle atrophy induced by type 2 diabetes (Bitar et al., 2018), hyperhomocysteinemia (Majumder et al., 2018), ventilator (Ichinoseki-Sekine et al., 2021), cardiotoxin (Zhang et al., 2021), and denervation (Kuo et al., 2015) by attenuating inflammation, oxidative stress and protein degradation. The present study demonstrated that treatment with either slow (GYY4137) or rapid (NaHS) H_2_S releasing donors protected mice against immobilization-induced muscle atrophy. In addition, we provided several line of evidence that both GYY4137 and NaHS inhibited immobilization-induced inflammation and oxidative stress in skeletal muscles as evidenced by decreases in pro-inflammatory factors, leukocyte infiltration, H_2_O_2_ level, 8-OHdG immunoreactivity and increases in anti-oxidative biomarker T-AOC. These findings suggest that the inhibitory effects of H_2_S on immobilization-induced inflammatory response and oxidative stress may at least partially be responsible for its protective effects against skeletal muscle atrophy.

Muscle fibrosis characterized by increased collagen deposition in the extracellular matrix is the main cause of reduced muscle extensibility and decreased range of motion in immobilization-induced skeletal muscle atrophy (Maezawa et al., 2017). Previous studies have shown that the accumulation of leukocytes, which induces the differentiation of fibroblasts into myofibroblasts via IL-1β/TGF-β signaling, plays an important role during the pathogenesis of immobilization-induced muscle fibrosis (Honda et al., 2015). On the other hand, reactive oxygen species (ROS) has been shown to mediate TGF-β1-induced fibroblast activation and myofibroblast differentiation in various tissues (Cucoranu et al., 2005; Tsubouchi et al., 2017). Our previous study has found that H_2_S alleviates renal injury and fibrosis in response to unilateral ureteral obstruction by attenuating macrophage infiltration (Zhou et al., 2020). In addition, H_2_S donors are also found to alleviate bleomycin-induced lung fibrosis (Du et al., 2019), transverse aortic constriction-induced myocardial fibrosis (Liu et al., 2021) and blunt contusion-induced skeletal muscle fibrosis (Zhao L et al., 2020) by attenuating oxidative stress. Consistent with these findings, the present study found that both GYY4137 and NaHS significantly decreased immobilization-induced leukocyte infiltration and oxidative stress, reduced the production of IL-1β and TGF-β in skeletal muscles, thus alleviating immobilization-induced muscle fibrosis.

H_2_S donors have been found to exert either pro-inflammatory or anti-inflammatory effects. This discrepancy is at least partially due to the concentration and releasing rate of different H_2_S donors (Bhatia et al., 2006). GYY4137 releases H_2_S slowly over a period of hours, thus maintaining a stable and sustained H_2_S concentration after injection *in vivo* (Li et al., 2009). In contrast, NaHS gives a rapid bolus of H_2_S in aqueous solutions (De Preter et al., 2016). Whiteman et al. have compared the effects of GYY4137 and NaHS on the release of inflammatory mediators in macrophages in the same concentration range (Whiteman et al., 2010). GYY4137 concentration-dependently inhibits LPS-induced release of proinflammatory mediators, whereas NaHS elicits a biphasic effect on macrophages and, at high concentrations, increases the synthesis of proinflammatory cytokines (Whiteman et al., 2010). Similar results are documented in vivo studies. GYY4137 at a dose of 50 mg/kg exerts anti-inflammatory effects in animal models of endotoxic shock (Li et al., 2009), joint inflammation (Li et al., 2013) and experimental necrotizing enterocolitis (Drucker et al., 2019). Administration of NaHS at high dose of 10 mg/kg has been shown to induce lung inflammation by itself, and also to worsen CLP-induced lung inflammation (Zhang et al., 2006). However, administration of NaHS at low dose of 0.5–2 mg/kg exerts anti-inflammatory effects in animal models of ovalbumin-induced airway inflammation (Benetti et al., 2013), homocysteine-induced neuro-inflammatory conditions (Kumar and Sandhir, 2019) and blunt contusion-induced skeletal muscle atrophy (Zhao L et al., 2020). In consistent with these studies, our findings showed that both GYY4137 (50 mg/kg) and NaHS (20 μM/kg or 1.12 mg/kg) significantly inhibited immobilization-induced inflammation in skeletal muscles.

Skeletal muscle atrophy occurs as the result of an imbalance between anabolic and catabolic processes (Wall et al., 2013a). In a variety of muscle-wasting conditions such as cancer, diabetes, sepsis, starvation and disuse, a primary driver of muscle atrophy is thought to be an increase in protein degradation via the ubiquitin–proteasome pathway (Kandarian and Jackman, 2006; Cohen et al., 2015). MuRF1 and atrogin-1 have been well-recognized as two muscle-specific E3 ubiquitin ligases that are transcriptionally increased in skeletal muscle under atrophy-inducing conditions (Komatsu et al., 2018). In the present study, we found that immobilization-induced mRNA expressions of MuRF1 and atrogin-1 in skeletal muscles were significantly decreased by administration of GYY4137 and NaHS, suggesting that H_2_S treatment inhibited MuRF1 and atrogin-1 expression in the transcriptional level. Notably, H_2_S can modify target proteins by S-sulfhydration at Cys residues (Lu et al., 2020), which also serves as an important signaling mechanism by H_2_S. Lu et al. recently report that H_2_S treatment results in MuRF1 S-sulfhydration at the site of Cys44, thus preventing the degradation of skeletal muscle and ameliorating skeletal muscle atrophy in diabetic mice (Lu et al., 2020). The possibility that H_2_S-mediated post-translational modification of muscle-specific E3 ubiquitin ligases may contribute to the protective effects of H_2_S against immobilization-induced muscle atrophy merits further investigation.

In addition, NRF2 is a ubiquitous master transcription factor that regulates antioxidant response element (ARE)-mediated expression of over 200 antioxidant enzymes and cytoprotective proteins (He et al., 2020). Kelch-like ECH-associated protein 1 (Keap1) acts as a negative regulator of NRF2. H_2_S has been reported to s-sulfhydrated Keap1 at cysteine-151 and induced NRF2 dissociation from Keap1, thus enhancing NRF2 nuclear translocation and transactivating the target genes of NRF2 (Yang et al., 2013). NRF2 signal is found to be down-regulated in denervation-induced skeletal muscle atrophy (Kuo et al., 2015). Baumfalk et al. have reported that NRF2 contributes to the protective effect of H_2_S against oxidative stress in a prostate cancer-induced cardiac atrophy model (Baumfalk et al., 2021). Consistent with these studies, the present study demonstrated significant decreases in NRF2 expression and transcriptional levels of NRF2 downstream target genes in IM-induced atrophied skeletal muscle. Furthermore, both slow (GYY4137) and rapid (NaHS) H_2_S releasing donors profoundly reversed IM-induced downregulation of NRF2 and its downstream anti-oxidant target genes in skeletal muscle. These findings suggest that H_2_S may protect against immobilization-induced skeletal muscle atrophy, at least partly, by activating NRF2 signaling pathway.

Meanwhile, AKT regulates both protein synthesis and degradation, respectively, via the kinases mammalian target of rapamycin (mTOR) and the transcription factors of the FoxO family (Bonaldo and Sandri, 2013). On one hand, AKT enhances protein synthesis of skeletal muscle by activating AKT/mTOR and downstream signaling molecules as reflected by the phosphorylation of S6 ribosomal protein (S6) and eIF4E-binding protein (4E-BP1) (You et al., 2015). On the other hand, AKT promotes the phosphorylation of FoxO1 and consequently decreases the expression of two degradation-associated proteins, namely, MuRF1 and atrogin-1 (Sandri et al., 2004). Bitar et al. have revealed that rat diabetic atrophied muscles exhibit a state of depressed AKT activation. H_2_S therapy increases AKT activity and levels of myostatin in diabetic skeletal muscle (Bitar et al., 2018). Similarly, we found that immobilization decreased phosphorylated AKT levels in TA muscles. However, either GYY4137 or NAHS had no significant effect on AKT phosphorylation in TA muscles exposed to immobilization. The discrepancy between our finding and those of Bitar et al. may be attributed to a lot of factors, such as muscle atrophy model differences and species and genus diversity of laboratory animals, which should be further investigated in the future research.

## Conclusion

The present study demonstrated for the first time that treatment with either slow (GYY4137) or rapid (NaHS) H_2_S releasing donors protected mice against immobilization-induced muscle fibrosis and atrophy. In addition, both GYY4137 and NaHS inhibited immobilization-induced inflammation and oxidative stress in skeletal muscles. These findings suggest that the beneficial effects of H_2_S on immobilization-induced skeletal muscle atrophy may be due to both the anti-inflammatory and anti-oxidant properties of H_2_S, thus supporting the therapeutic potentials of H_2_S donors in patients with disuse-associated muscle atrophy.

## Data Availability

The raw data supporting the conclusions of this article will be made available by the authors, without undue reservation.
