# Supplementary material for: H2S Protects Against Immobilization-Induced Muscle Atrophy via Reducing Oxidative Stress and Inflammation
Source: Front Physiol. 2022 Apr 6;13:844539. doi: 10.3389/fphys.2022.844539 (PMC9019569; doi:10.3389/fphys.2022.844539)

Supplementary figure legends

S-Figure 1. NAHS at the dose of 20 µmol/kg had a better protective effect than 50 µmol/kg in maintaining muscle mass and decreasing muscle atrophy related genes expressions. Mice were subjected to IM and treated with NaHS (20 µmol/kg or 50 µmol/kg twice a day, ip) for two weeks. The control and IM groups received identical doses of saline for 2 weeks. a, TA, GAS, and QUA muscle weights in control, IM, IM + 20 µmol/kg NaHS mice, and IM + 50 µmol/kg NaHS mice (n = 8 in each group). b, Expression of MuRF1 and atrogin-1 in control, IM, IM + 20 µmol/kg NaHS mice, and IM + 50 µmol/kg NaHS mice (n = 8 in each group). Data represent means ± SEM. *p < 0.05, **p < 0.01, ***p < 0.001.

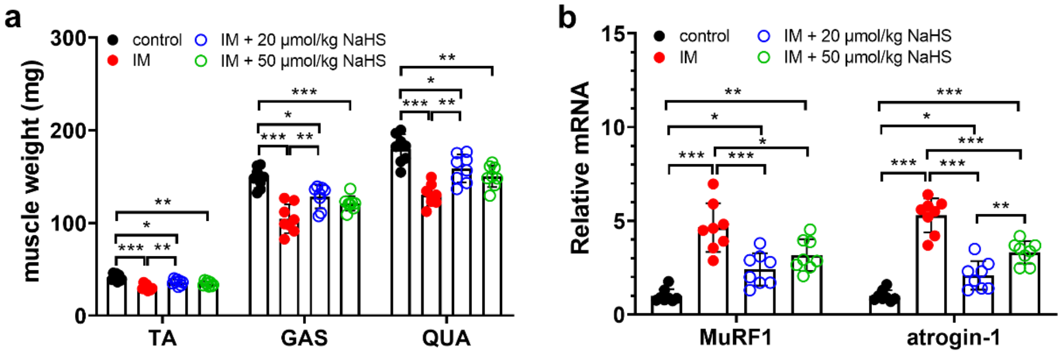

Supplement: Supplementary file 1 [file Image1.pdf]
